# Shikonin inhibits MRSA biofilm formation to alleviate periprosthetic joint infection

**DOI:** 10.3389/fphar.2026.1739888

**Published:** 2026-03-06

**Authors:** Shangyi Liu, Haoran Zhang, Xi Zheng, Xiaoqin Mou, Zhongbao Wu, Lili Zou, Kangquan Shou, Xiaowen Liu

**Affiliations:** 1 Department of Orthopedics, The First College of Clinical Medical Science and Yichang Central People’s Hospital, China Three Gorges University, Yichang, China; 2 Hubei Key Laboratory of Tumor Microenvironment and Immunotherapy and College of Basic Medical Sciences, China Three Gorges University, Yichang, China; 3 Yichang Key Laboratory of Infection and Inflammation, College of Basic Medical Sciences, China Three Gorges University, Yichang, China

**Keywords:** shikonin (SKN), periprosthetic joint infection (PJI), methicillin-resistant *Staphylococcus aureus* (MRSA), biofilm formation, cysteine metabolism

## Abstract

**Objective:**

To alleviate periprosthetic joint infection (PJI) with methicillin-resistant *Staphylococcus aureus* (MRSA), shikonin (SKN) had been used to intervene the biofilm formation of MRSA *in vivo* and *in vitro*, which provides theoretical support and practical foundation for SKN as a novel drug against drug-resistant bacterial infection.

**Methods:**

The rat model of periprosthetic joint infection was established, utilizing techniques such as scanning electron microscopy and pathology test to evaluate the MRSA inhibitory of bacterial load and biofilm formation effects of SKN. The minimum inhibitory concentration (MIC) and minimum bactericidal concentration (MBC) experiments were conducted to assess the antibacterial activity of SKN. The crystal violet staining method was employed to evaluate the effects of SKN on MRSA biofilm formation and eradication. Transcriptomic and amino acid metabolomics analyses were used to investigate the mechanism of SKN inhibition in MRSA biofilm formation. Total thiol detection was used to assess the impact of SKN on the intracellular cysteine levels in MRSA. Finally, MIC and crystal violet staining were used to evaluate the antibacterial effects and biofilm eradication efficacy of SKN against clinical MRSA strains.

**Results:**

*In vivo* experimental results demonstrated that high doses of SKN significantly reduced the biofilm formation in MRSA PJI in rats, improved local inflammatory responses, and promoted tissue repair. Observations using scanning electron microscopy confirmed that SKN effectively inhibited the formation of biofilms on implant surface. MIC experiments revealed that the lowest inhibitory concentration of SKN was 70 μM, indicating significant antibacterial activity, although no direct bactericidal effects were observed. Results of crystal violet staining showed that SKN could significantly inhibit biofilm formation of MRSA at sublethal concentrations and exhibited efficacy of biofilm removal. Transcriptomic and acid amino metabolomic analyses prompted that the inhibition of MRSA biofilm formation by SKN might be related to regulate the cysteine metabolism in MRSA. Total thiol detection was used to validate the omics findings *in vitro*. Finally, SKN intervention in MRSA clinical strains showed that the SKN could inhibit MRSA clinical strains and remove biofilm.

**Conclusion:**

SKN inhibits MRSA by suppressing biofilm formation, effectively alleviating periprosthetic joint infection by MRSA, and the mechanism of SKN antibacterial activity may be related to regulate the cysteine metabolism in MRSA.

## 1 Introduction

Despite continuous advancements in perioperative antibiotic prophylaxis and aseptic techniques, the incidence of periprosthetic joint infection (PJI) remains on the rise (Feng et al., 2020; Izakovicova et al., 2019; Karczewski et al., 2019). With the aging population, the global number of joint replacement surgeries will exceed 3.8 million annually by 2030, with infection cases potentially reaching 266,000 per year (Bozic and Ries, 2005; Kurtz et al., 2008; Kurtz SM. et al., 2007; Kurtz S. et al., 2007). The key challenge in treating PJI lies on the biofilm formation by resistant bacterial strains on the surface of implants, which hinder the efficacy of antibiotics in achieving bactericidal or bacteriostatic effects (Wimmer et al., 2020; Charalambous et al., 2022; Froschen et al., 2022a). *Staphylococcus aureus* species account for 70% of PJIs, among which methicillin-resistant *S. aureus* (MRSA) infection represents the most severe and difficult-to-treat case in post-arthroplasty (Tsai et al., 2019; Froschen et al., 2022b).

Currently, traditional antibiotics such as ß-lactams and vancomycin exhibit limited efficacy against bacteria within biofilms, and the issue of antibiotic resistance is becoming increasingly (Alekshun and Levy, 2007; Orsi et al., 2011). Therefore, the development of novel anti-biofilm agents is urgently needed. Shikonin (SKN), a naphthoquinone compound extracted from plants in *Boraginaceae* family, has been shown to possess multiple biological activities, including antibacterial, anti-inflammatory, and wound-healing effects in previous studies. Notably, it demonstrates potential inhibitory activity against MRSA and vancomycin-resistant *Enterococcus* species (Mei et al., 2020; Xiaojing et al., 2023; Shen et al., 2002).

**FIGURE 1:**
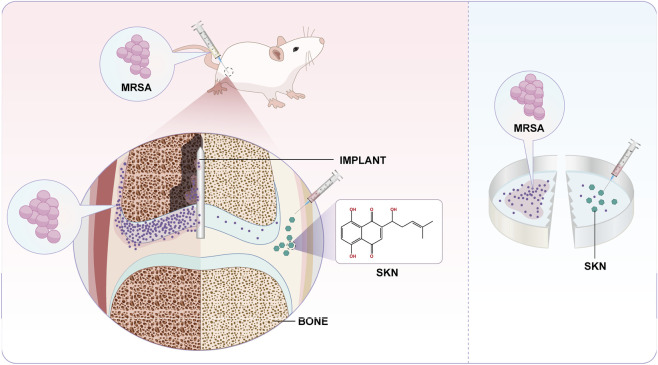
SKN can effectively inhibit bacterial colonization and biofilm formation in MRSA-related periprosthetic joint infection both *in vitro* and *in vivo*.

Based on that, this study focuses on evaluating the efficacy of SKN in inhibiting MRSA infection and biofilm formation in the rat periprosthetic joint infection model. Additionally, it employs integrated transcriptomic and metabolomic analyses to preliminarily elucidate the molecular mechanisms underlying SKN anti-biofilm activity. The findings aim to provide a theoretical basis for developing SKN as a novel anti-biofilm therapeutic agent (Figure 1).

## 2 Materials and methods

### 2.1 MRSA strains

The MRSA strain USA300 was kindly provided by Professor Sun from University of Science and Technology of China, which is maintained by our research team. The clinical MRSA strains (MRSA-602, MRSA-131, MRSA-738, MRSA-996) were collected from hospital in previous studies by our group. The MRSA strains were cultured on LB agar plates, and single colonies were picked and inoculated into 5.0 mL of LB. The cultures were incubated with shaking at 37 °C and 180 rpm for 12 h. The next day, 1.0 mL of bacterial suspension was transferred into 100 mL LB and incubated at 37 °C with shaking at 180 rpm for 3 h to obtain logarithmic-phase MRSA bacteria, which were then used for subsequent experiments.

### 2.2 Experimental animals

Thirty-five SD rats (male, 8 weeks old, weighing 300 ± 20 g, SPF) were used in this study. All animals were housed in ventilated, sterilized cages maintained at 25 °C ± 2 °C with a humidity of 55% ± 5%. The rats followed a 12-h light/dark cycle and had free access to standard laboratory chow and water. The experimental animal test was supplied by the Animal Experiment Center of Three Gorges University and approved by the Animal Ethics Committee of the Animal Experiment Center at Three Gorges University (2024050U1).

### 2.3 Reagents and materials

All reagents and commercial kits used in this study were purchased from official suppliers with complete source information: Shikonin (SKN) was obtained from Macklin Biochemical Technology Co., Ltd. (Shanghai, China); Cefazolin (CEF) from Chengdu Brilliant Pharmaceutical Co., Ltd. (Chengdu, China); Vancomycin (VAN) from North China Pharmaceutical Group Co., Ltd. (Shijiazhuang, China); methanol and acetonitrile from Merck KGaA (Darmstadt, Germany); Trizol reagent for RNA extraction from Invitrogen Corporation (Carlsbad, CA, United States); crystal violet from Solarbio Science and Technology Co., Ltd. (Beijing, China); glacial acetic acid from Feijing Biotechnology Co., Ltd. (Shanghai, China); TruSeq™ RNA Sample Preparation Kit from Illumina, Inc. (San Diego, CA, United States); Glutathione (GSH) detection kit from Nanjing Jiancheng Bioengineering Institute (Nanjing, China); 4% paraformaldehyde, Hematoxylin-Eosin (H&E) staining kit, and Gram staining kit from Biosharp Biotechnology Co., Ltd. (Nanjing, China); anti-rat TNF-α antibody and anti-rat IL-1β antibody (corrected from IL-6 to align with experimental results) from Univin Biotechnology Co., Ltd. (Suzhou, China); orthopedic-grade Kirschner wires from Yuyue Medical Equipment and Supply Co., Ltd. (Zhenjiang, China); NanoDrop 2000 spectrophotometer from Thermo Fisher Scientific (Waltham, MA, United States); Agilent 2100 Bioanalyzer from Agilent Technologies, Inc. (Santa Clara, CA, United States); cell lysis buffer for GSH detection was included in the GSH detection kit (Nanjing Jiancheng Bioengineering Institute, Nanjing, China); LB agar and LB broth from BD Difco (Franklin Lakes, NJ, United States); and phosphate-buffered saline (PBS) from Solarbio Science and Technology Co., Ltd. (Beijing, China).

### 2.4 Minimum inhibitory concentration and minimum bactericidal concentration

The concentration of logarithmic-phase MRSA bacterial suspension was determined by dual quantification via optical density (OD) measurement at 600 nm and serial dilution plating on LB agar, followed by serial dilution with sterile LB medium to adjust to a final working concentration of 1.0 × 10^6^ CFU/mL. In a 96-well plate, each well was filled with 100 µL of SKN (molecular weight: 288.3 g/mol), solution at varying concentrations. Subsequently, 100 µL of pre-diluted MRSA bacterial suspension was added to each well, resulting in a total liquid volume of 200 µL per well. The plate was incubated at 37 °C for 24 h. During the incubation period, the OD_600_ was measured using a microplate reader at intervals of 2, 4, 8, 16, and 24 h to assess bacterial growth. After determining the MIC, 10 µL of culture medium was taken from wells showing no apparent bacterial growth and spread onto LB agar plates without drugs. These plates were then incubated in a 37 °C constant temperature incubator for 24 h to observe colony growth. By counting the number of colonies, the lowest drug concentration that showed no colony growth on the medium was determined as the minimum bactericidal concentration (MBC).

### 2.5 Crystal violet staining

Inhibition assay: 200 µL of diluted bacterial suspension was added to each well in a 96-well plate, followed by the addition of the test compound. The plate was incubated with SKN at 37 °C for 24 h to promote biofilm formation. After incubation, the liquid in each well was carefully discarded, and unattached planktonic bacteria were gently rinsed with PBS buffer for three times. Subsequently, biofilms were fixed with 99% methanol for 15 min. The supernatant was then removed, and biofilms were stained with 2% crystal violet for 5 min. The plate was rinsed with PBS, and the dye was dissolved using 200 µL of glacial acetic acid. OD_595_ was measured for each well using a microplate reader.

Clearance Assay: 200 µL of diluted bacterial suspension was added to each well in a 96-well plate at 37 °C for 12 h to promote biofilm formation. Subsequently, drugs at different concentrations were added to each well according to the experimental design, and the plate was incubated statically at 37 °C for another 12 h. After drug treatment, the OD_595_ was measured using a microplate reader.

### 2.6 Transcriptomics

For transcriptomic analysis, MRSA samples were collected from the MIC-dose SKN treatment group (n = 5) and the solvent control group (n = 3) at 24 h post-incubation. Briefly, 1 mL of bacterial culture from each well was harvested by centrifugation at 8,000 rpm for 5 min at 4 °C under sterile conditions. The bacterial pellets were washed twice with ice-cold sterile PBS to remove residual LB medium, SKN, and DMSO, and then immediately resuspended in 1 mL of Trizol reagent to inhibit RNA degradation. The resuspended samples were vortexed for 30 s to fully lyse bacterial cells and stored at −80 °C until total RNA extraction. Total RNA was extracted using Trizol reagent, and RNA purity was determined using the NanoDrop 2000 spectrophotometer. RNA integrity was assessed using the Agilent 2100 Bioanalyzer, with RNA samples requiring a RIN value of ≥8.0 for subsequent experiments. For each sample, 2 µg of total RNA was used to construct sequencing libraries with the TruSeq™ RNA Sample Preparation Kit. After passing library quality control, paired-end 150 bp high-throughput sequencing was performed on the Illumina NovaSeq 6000 platform. Low-quality and non-informative data were filtered out, followed by COG enrichment analysis.

### 2.7 Metabolomics

Metabolomic analysis was performed to identify differential metabolites between SKN-treated and control MRSA groups: 0.05 g of frozen MRSA bacterial pellets (harvested by centrifugation at 8,000 rpm for 5 min at 4 °C, consistent with transcriptomic sample collection) was weighed and mixed with 500 µL of pre-cooled (−20 °C) 70% methanol/water (v/v), followed by vortexing at 2500 rpm for 3 min and ultrasonic extraction (4 °C, 15 min) to release metabolites, centrifugation at 12,000 rpm for 10 min at 4 °C, protein precipitation of 300 µL supernatant at −20 °C for 30 min, re-centrifugation under the same conditions, and filtration of the final 200 µL supernatant through a 0.22 μm membrane before storage at −80 °C for LC-ESI-MS/MS analysis; the analysis was conducted on an Agilent 1290 UHPLC coupled with a 6470 Triple Quadrupole mass spectrometer using a Waters ACQUITY UPLC HSS T3 column (2.1 mm × 100 mm, 1.8 μm) at 40 °C, with a mobile phase of 0.1% formic acid in water (A) and acetonitrile (B), a flow rate of 0.3 mL/min, and gradient elution (0–2 min: 5% B; 2–10 min: 5%–95% B; 10–15 min: 95%–5% B), while an ESI source was used in positive/negative modes (capillary voltage: 3.5/3.2 kV, mass scan range: m/z 50–1000) with multiple reaction monitoring (MRM); raw data were processed via Progenesis QI 2.3 (median normalization, peak alignment/picking), metabolites were identified using HMDB/KEGG databases, differential metabolites were screened by VIP ≥1.0 (PLS-DA) and P < 0.05 (one-way ANOVA), and KEGG pathway enrichment analysis was performed with MetaboAnalyst 5.0 (FDR <0.05).

### 2.8 Determination of glutathione

Collect MRSA samples from the SKN treatment group and the control group. Resuspend the samples in PBS, centrifuge, and discard the supernatant. Add cell lysis buffer to the samples. Perform ultrasonic disruption to lyse the cells, centrifuge, and transfer the supernatant into a 96-well plate. Set up blank wells, standard wells, and sample wells. Mix the samples with DTNB and incubate at room temperature in the dark for 10 min. Measure the OD_412_ of each well using a microplate reader. Calculate the glutathione (GSH) content in each well based on the standard curve provided by the assay kit, correcting for protein concentration.

### 2.9 Antibacterial effect in rat model of PJI

#### 2.9.1 Preparation of MRSA

The MRSA (USA300) strain was stored in a −80 °C freezer. After thawing, the MRSA bacterial suspension was streaked onto the surface of an LB agar medium and incubated overnight at 37 °C for 18–24 h. A single colony was then selected and transferred to LB medium. Once the bacteria reached the logarithmic growth phase, the culture was centrifuged and washed twice with PBS. Finally, the bacteria were resuspended in PBS to achieve a bacterial suspension concentration of 2 × 10^6^ cfu/mL.

#### 2.9.2 Experimental grouping

The study included the following groups: the low-dose SKN group (LD-SKN; n = 5), which received intra-medullary injection of MRSA and intra-articular injection of SKN at 1.5 mg/kg; the high-dose SKN group (HD-SKN; n = 5), which received intra-medullary injection of MRSA and intra-articular injection of SKN at 3.0 mg/kg; the control group (Control; n = 5), which received intra-medullary injection of MRSA and intra-articular injection of SKN solvent; the Cefazolin group (CEF; n = 5), which received intra-medullary MRSA injection, with 30 mg/kg cefazolin layered uniformly into the surgical field prior to closure to simulate the prophylactic use of antibiotics during clinical knee replacement surgery; the Vancomycin group (VAN; n = 5), which received intra-medullary MRSA injection, with 10 mg/kg vancomycin layered into the surgical field before wound closure to simulate prophylactic antibiotic use; the model group (Model; n = 5), which received intra-medullary MRSA injection and intra-articular injection of normal saline; and the blank group (Blank; n = 5), which underwent the same surgical procedure without MRSA injection, with Kirschner wire insertion followed by immediate wound closure. All rats were euthanized on day 21, and various assessments were performed on each subject. The doses of SKN were determined based on preliminary pre-experiments, which were converted from its *in vitro* MIC against MRSA and confirmed to achieve antibacterial efficacy while causing no obvious toxicity or adverse effects in rats.

#### 2.9.3 Surgical procedure

SD rats were anesthetized using inhaled isoflurane. The fur in the surgical area was trimmed with clippers, and the area was disinfected with povidone-iodine and 75% medical alcohol. An incision was made on the medial side of the knee joint, adjacent to the patella, to access the distal femur. The quadriceps-patellar complex was displaced laterally to locate the intercondylar fossa of the femur. A Kirschner wire was used to pre-drill the medullary cavity, and 50 µL of MRSA bacterial solution was injected into the cavity using a syringe. An orthopedic-grade Kirschner wire was then inserted, leaving 1 mm in the joint space to facilitate later removal. The patellar-quadriceps complex was repositioned to the midline, and the fascia was closed with absorbable surgical sutures. Each group of rats received corresponding interventions. An X-ray examination was conducted immediately after surgery to confirm the position of the prosthesis, and the surgical incision was closed. Postoperatively, 5 mg/kg of meloxicam was administered subcutaneously daily to alleviate pain.

#### 2.9.4 Microbial cultivation

On the 21st day post-surgery, the rats were euthanized, and the Kirschner wires, along with the femoral canal bone fragments and soft tissue, were collected. These samples were homogenized in sterile saline. After ultrasonic treatment, they were vortexed again for an additional 30 s. A suspension solution (1:10) was prepared and inoculated onto LB agar plates, followed by incubation at 37 °C for 18–24 h (Lopez-Torres et al., 2020).

#### 2.9.5 Histological analysis

The rat knee joints were preserved in 4% paraformaldehyde, followed by decalcification and paraffin embedding. Sagittal sections with a thickness of 4 mm were prepared and subsequently stained using hematoxylin-eosin (H&E) and Gram staining for histological analysis. Observations were conducted under an optical microscope. Immunohistochemical (IHC) analysis was performed, utilizing antibodies targeting tumor necrosis factor-alpha (TNF-ɑ, Univin) and interleukin-1 (IL-1, Univin).

#### 2.9.6 Scanning electron microscopy

On day 21 post-surgery, the implants were carefully extracted to preserve biofilm integrity, then fixed with 2.5% glutaraldehyde, dehydrated through a graded ethanol series, dried by critical point drying, and sputter-coated with gold. Observations and imaging of the implant surface biofilm—with 3 random non-overlapping fields per sample imaged at magnifications of ×30, ×500, and ×1000 to assess biofilm formation and morphological characteristics—were performed using a scanning electron microscope at an accelerating voltage of 10 kV.

### 2.10 Statistical analysis

The results are presented as mean ± standard deviation (SD). Statistical analyses were conducted using GraphPad Prism 9. Differences between groups were assessed using one-way ANOVA. *, *P* < 0.05; **, *P* < 0.01. Sample size calculation was performed using G*Power 3.1 software based on preliminary experiments. For animal experiments (*in vivo* PJI model), the effect size (Cohen’s d) of SKN on MRSA biofilm inhibition was determined as 1.2 (α = 0.05, power = 0.8), indicating that 5 rats per group were sufficient to detect significant differences. For *in vitro* experiments and omics analyses, a sample size of 3–5 replicates per group was selected to achieve a statistical power of ≥80%, consistent with standard practices for microbiological and multi-omics studies. Effect sizes (η^2^ for one-way ANOVA) were calculated to assess the practical significance of group differences. For overlapping analyses of omics data, hypergeometric tests were used to verify the statistical significance of co-enriched pathways, with false discovery rate (FDR) correction to control for multiple comparisons.

## 3 Results

### 3.1 SKN alleviated periprosthetic joint infection by MRSA in rat models

**FIGURE 2:**
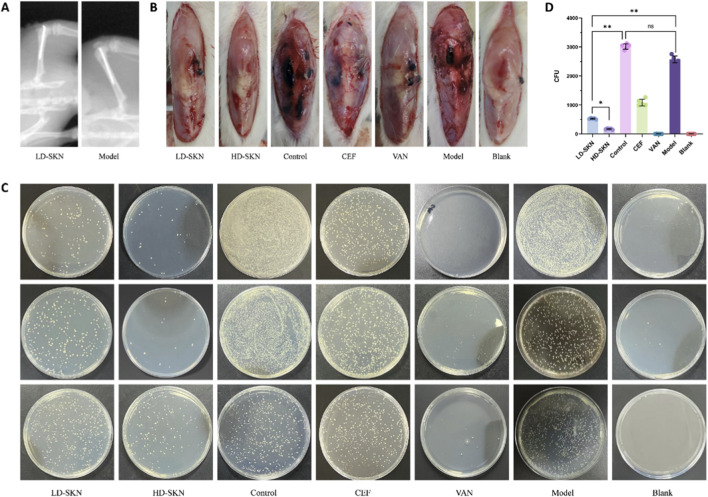
Antibacterial effects of SKN against MRSA *in vivo* **(A)** the insertion of Kirschner wires into the knee joints of rats; **(B)** The condition of the local surgical area of the knee joint after skin incision in euthanized rats at 21 days post-operation, with similar results observed within each group; **(C)** Bacterial culture plates demonstrating results, with similar findings within each group; **(D)** Bar graph depicting colony counts per mL, with data presented as mean ± SD. (n = 5; ns: *P* > 0.05, *P < 0.05, **P < 0.01).

**FIGURE 3:**
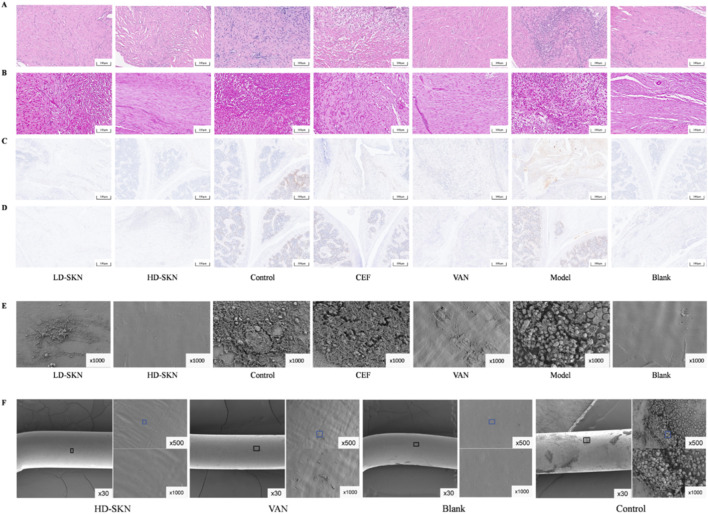
SKN exhibited the anti-MRSA ability and inhibited MRSA biofilm formation **(A)** H&E staining of the femur tissues which established PJI model; **(B)** Gram staining of femur tissues which established PJI model; IHC of IL-1ß **(C)** and TNF-ɑ **(D)** in femur tissues which established PJI model; **(E)** the biofilm on the surface of implant under SEM; **(F)** Overall biofilm appearance under SEM with a specific area (black box) at different magnifications.

To evaluate the inhibitory effects of SKN on periprosthetic joint infection by MRSA, we established the rat models (Figure 2A) with SKN treatment for a 21-day observation (Figure 2B). On the 21st day treatment, bacterial cultures were performed on samples from the rat knee joints, surrounding prosthetic tissues, and bone (Figures 2C,D). The results showed that, compared to the other groups, SKN demonstrated varying degrees of antibacterial effects as well as VAN group, with the efficacy positively correlated with concentration. Then, histopathological analyses were employed to observe the effect of SKN. H&E staining sections (Figure 3A) and Gram staining (Figure 3B) were detected in femur tissues. In HD-SKN group, the inflammatory cell and gram-positive bacteria (stained blue) were significantly reduced, comparing with other groups. Furthermore, IHC results (Figures 3C,D) indicated that the expression levels of IL-1ß and TNF-ɑ in the treatment groups were significantly lower than those in the control group. These histological findings further demonstrate that SKN possesses *in vivo* bioactivity to inhibit MRSA induced periprosthetic joint infection.

### 3.2 SKN inhibition of biofilm formation on the surface of implants

On the 21st day post-surgery, implants were retrieved from the rats and the surface of implants was visualized using scanning electron microscopy (SEM). In Model group, a distinct biofilm had formed on the implant surface on day 21, which indicated that it was developed infection successfully in the rat periprosthetic joint infection model we constructed, with bacteria forming biofilms on the implant surface, consistent with the biofilm formation observed in patients with post-joint replacement infections (Darouiche, 2004; Zimmerli et al., 2004; Del Pozo and Patel, 2009; Trampuz and Widmer, 2006). In contrast, no biofilm formation was observed on the implant surface from SKN groups and VAN group, which appeared nearly identical to the Blank group (Figures 3E,F), that indicated SKN effectively inhibited biofilm formation, demonstrating the potential application value in anti-infective therapy.

### 3.3 Eliminated MRSA biofilm formation *In vitro* by SKN

**FIGURE 4:**
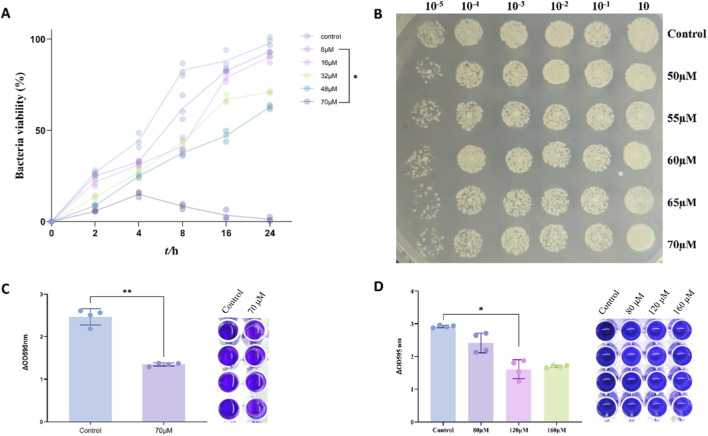
The antibacterial effect of SKN against MRSA *in vitro*. The MIC **(A)** and MBC **(B)** of SKN against MRSA; The inhibitory effect of SKN on MRSA biofilm formation **(C)** clearance **(D)** and were assessed using crystal violet staining. All *in vitro* experiments were independently repeated 3 times. (n = 3; mean ± SD; *P < 0.05, **P < 0.01).

Due to the antibacterial and biofilm formation inhibition effect of SKN *in vivo*, the MIC and MBC of SKN against MRSA were detected *in vitro*. The results of the MIC test showed that MRSA growth was significantly inhibited by SKN as the concentration increased (Figure 4A). The experiments determined the MIC of SKN to be 70 μM, at which bacterial activity was notably suppressed, demonstrating the antibacterial activity of SKN against MRSA. The results of the MBC assay indicated that there was no significant difference in the colony count of MRSA following treatment with SKN at various concentrations (Figure 4B). SKN did not exhibit bactericidal activity against MRSA, with its primary effect being bacteriostatic instead. Thus, SKN exerts a potent inhibitory effect on MRSA but lacks direct bactericidal activity. Quantitative analysis of biofilms using crystal violet staining (Figure 4C) showed that the formation of MRSA biofilm was significantly reduced in the SKN-treated group compared to the control group. At SKN sub-lethal concentration in MRSA, biofilm formation was inhibited. Figure 4D further illustrated the biofilm-clearing effect of SKN on pre-formed biofilms. The experimental results demonstrated that SKN, at sub-lethal concentration, not only inhibited biofilm formation but also exhibited a certain degree of clearing activity against established biofilm of MRSA. These findings suggest that SKN is effective in both preventing biofilm formation and clearing pre-existing biofilm.

### 3.4 Biofilm clearance ability of SKN via inhibiting MRSA cysteine metabolisma

**FIGURE 5:**
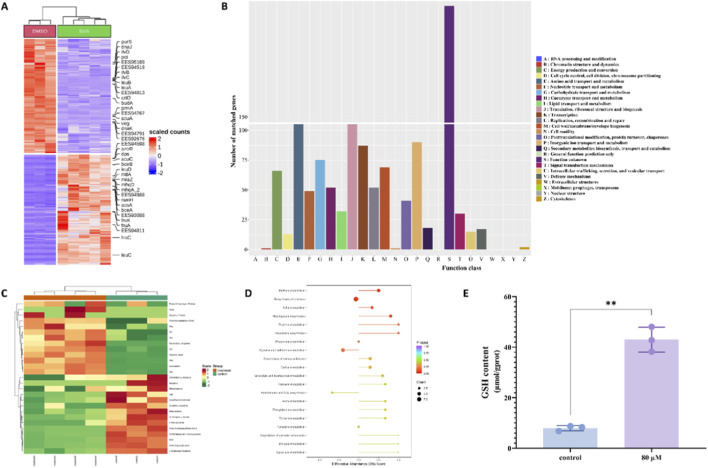
SKN regulated cysteine metabolism in MRSA. **(A)** Heatmap of differentially expressed genes between the SKN treatment group and the control group (a total of 2,136 genes), meeting the criteria of p-value <0.05 and |log2 FC| > 2, (n = 5 replicates for SKN group, n = 3 replicates for control group); **(B)** Functional category enrichment analysis of differentially expressed genes after SKN treatment using COG classification; each bar represents the number of differentially expressed genes corresponding to the respective functional category; **(C)** Heatmap of differential metabolites (n = 3); **(D)** Differential abundance score chart of KEGG metabolic pathways for differential metabolites; **(E)** Changes in GSH content in MRSA between the SKN treatment group and the control group. All omics and biochemical experiments were independently repeated 3 times. (mean ± SD; **P < 0.01).

To investigate the molecular mechanisms of SKN influences biofilm formation, we conducted transcriptomic analysis on MRSA samples from the SKN-treated group and the control group. The heatmap revealed that up to 40 genes exhibited significant changes in expression between the two groups (Figure 5A). Some genes involved in amino acid synthesis and metabolic pathways were downregulated in the SKN-treated group, suggesting that SKN might interfere with MRSA amino acid synthesis and metabolism processes. To gain a more comprehensive understanding of SKN effects on MRSA, we categorized all differentially expressed genes by function and performed functional enrichment analysis using COG classification (Figure 5B). The results indicated that amino acid metabolism in MRSA underwent significant changes following SKN treatment.

Subsequently, we employed amino acid metabolomics analysis to explore the specific pathways affected by SKN in MRSA amino acid metabolism. The results showed that, compared to the control group, 27 differential metabolites were identified in the SKN-treated group, with 14 metabolites significantly increased and 13 significantly decreased (Figure 5C). Further KEGG enrichment analysis revealed notable changes in the cysteine/methionine metabolism pathway (Figure 5D), which is associated with bacterial biofilm formation (Dinicola et al., 2014; Jochim et al., 2019).

To validate the changes observed in cysteine-related metabolic pathways from the omics analysis, we measured the GSH content in MRSA following SKN intervention. As shown in Figure 5E, the GSH content in MRSA was significantly elevated in the SKN-treated group compared to the control group. This result aligned with the trends observed in cysteine metabolism-related pathways in both transcriptomic and metabolomic analyses, indicating SKN potential regulatory effects on MRSA cysteine metabolism, which downregulated GSH synthesis.

### 3.5 SKN exhibited significant antibacterial activity against MRSA clinical strains

**FIGURE 6:**
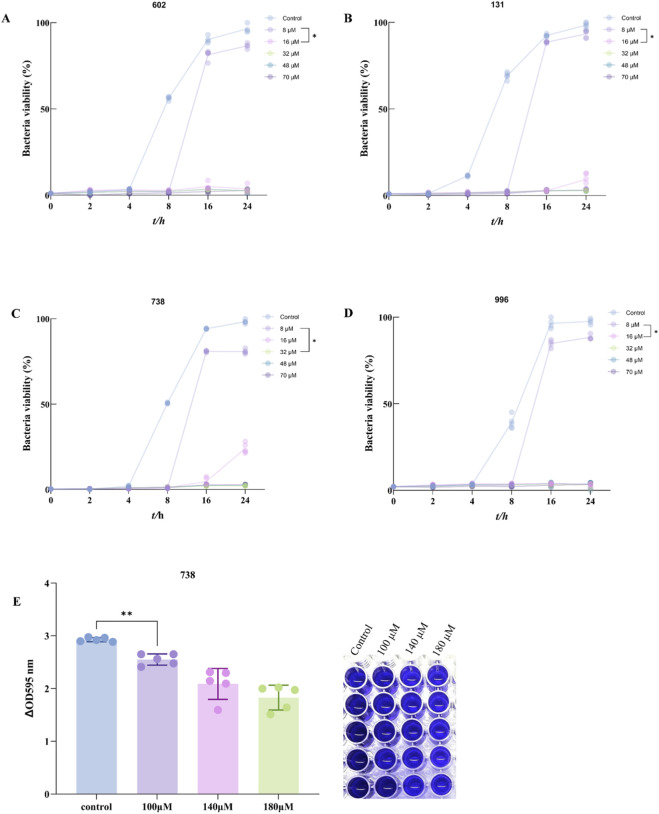
Antibacterial effect of SKN on MRSA clinical strains. **(A)** Bacterial survival rate of MRSA clinical strain 602 detected continuously for 24 h under different concentrations of SKN treatment; **(B)** Bacterial survival rate of MRSA clinical strain 131 detected continuously for 24 h under different concentrations of SKN treatment; **(C)** Bacterial survival rate of MRSA clinical strain 738 detected continuously for 24 h under different concentrations of SKN treatment; **(D)** Bacterial survival rate of MRSA clinical strain 996 detected continuously for 24 h under different concentrations of SKN treatment; **(E)** The biofilm removal effect of SKN in the MRSA clinical strain 738 was evaluated using crystal violet staining. (mean ± standard deviation). (n = 5,*P < 0.05,**P < 0.01).

To further evaluate the potential clinical application of SKN, we intervened with SKN in four clinically MRSA strains (MRSA-602, MRSA-131, MRSA-738, and MRSA-996) and measured the MIC. As shown in Figures 6A–D, the survival rates of all clinical MRSA strains significantly decreased with increasing SKN concentrations, indicating that SKN also exhibits good anti-MRSA activity against clinical strains. A randomly selected clinical strain (MRSA-738) was observed using crystal violet staining, which revealed that SKN effectively cleared the biofilm of MRSA-738 (Figure 6E).

## 4 Discussion

This study systematically evaluated the therapeutic efficacy of SKN in a rat model of PJI and demonstrated its ability to inhibit the formation of MRSA biofilms. Subsequently, through *in vitro* experiments, we confirmed that SKN can suppress MRSA biofilm formation and eradicate existing MRSA biofilms. The potential molecular mechanism is associated with SKN’s inhibition of the cysteine/methionine metabolic pathway in MRSA. Finally, we assessed the antimicrobial activity of SKN against clinical isolates, elucidating its potential as a therapeutic agent for bacterial biofilm infections.

The study results demonstrate that SKN significantly inhibits the formation of MRSA biofilms and, to some extent, can eradicate pre-formed biofilms, providing strong evidence for its potential development as an anti-biofilm therapeutic agent. This finding is consistent with previous research on the antibacterial activity of SKN (Han et al., 2023). The formation of MRSA biofilms is a major factor contributing to the difficulty in curing chronic infections, as these biofilms are composed of extracellular polymeric substances (EPS) produced by bacteria, which effectively resist the host immune system and antibiotic treatments (Wimmer et al., 2020; Charalambous et al., 2022). SKN enhances its antibacterial efficacy by inhibiting the production of EPS.

Like other plant-derived natural products, SKN functions as a versatile natural compound capable of inhibiting bacterial biofilm formation through multiple mechanisms. Studies have demonstrated that many plant extracts, such as tea polyphenols and curcumin, can interfere with biofilm formation by affecting bacterial adhesion, inhibiting the synthesis of extracellular polymeric substances, or disrupting quorum sensing signaling pathways (Schestakow et al., 2023; Rajasekar et al., 2021). SKN may inhibit MRSA biofilm formation through similar mechanisms. The results indicate that SKN exhibits a dose-dependent inhibitory effect on MRSA biofilm formation, interfering with key metabolic pathways or cellular signal transduction processes.

Notably, SKN not only inhibits the initial formation of biofilms but also has the ability to eradicate mature biofilms. Considering that bacteria within mature biofilms are often in a state of metabolic dormancy and exhibit strong resistance to antibiotics (Froschen et al., 2022a; Froschen et al., 2020; Darwich et al., 2021; Theng et al., 2020), SKN may increase the sensitivity of biofilms by activating the metabolism of dormant bacteria or disrupting their metabolic balance. Moreover, SKN demonstrates a clearing effect even at sub-lethal doses, potentially influencing the formation and stability of biofilms through multi-target actions.

In the rat PJI model, SKN demonstrated excellent anti-MRSA efficacy. Notably, in the high-dose group, it significantly inhibited biofilm formation, alleviated local inflammatory responses, and improved tissue repair conditions. Compared to the control group and the traditional antibiotic treatment group, SKN-treated tissues exhibited marked histopathological improvement. Considering that a major challenge in PJI treatment lies in the difficulty of antibiotics penetrating biofilms, leading to recurrent infections (Froschen et al., 2022a; Froschen et al., 2020; Darwich et al., 2021), the biofilm-clearing effect of SKN offers a novel approach to addressing this issue. Furthermore, it suggests that SKN could serve as a postoperative preventive or therapeutic strategy, showcasing unique advantages in the context of multidrug-resistant bacteria.

This study integrates multi-omics data with functional validation experiments to uncover the potential mechanism by which SKN inhibits MRSA biofilm formation through interference with the cysteine metabolic network. Although transcriptomic analysis did not detect significant changes in the expression of key cysteine synthesis genes (such as *CysK* and *CysM*), metabolomic data revealed a significant reduction in cysteine levels, accompanied by a marked increase in GSH synthesis following SKN treatment. These findings suggest that SKN may drive the rapid conversion of cysteine to GSH by accelerating cysteine metabolic pathways rather than directly regulating the expression of its synthesis-related genes. This redistribution of metabolic flux could lead to excessive depletion of the cysteine pool, thereby limiting the availability of critical thiol groups (such as disulfide-crosslinked extracellular polysaccharides) required for bacterial biofilm matrix synthesis. Ultimately, this disruption compromises the structural stability of the biofilm (Hufnagel et al., 2018).

As a core intracellular antioxidant molecule, the synthesis of GSH is highly dependent on the supply of cysteine. In this study, the increase in GSH may reflect a compensatory defense response of bacteria under SKN stress. However, the accompanying decrease in oxidized glutathione (GSSG) content suggests that SKN may lead to an imbalance in the GSH/GSSG ratio by inhibiting glutathione reductase activity or interfering with the NADPH regeneration system. This disruption of redox homeostasis may further exacerbate the metabolic stress in bacteria. Studies have shown that excessive consumption of GSH or impaired GSSG reduction capacity can trigger the accumulation of reactive oxygen species (ROS), which can significantly weaken biofilm formation by inhibiting extracellular polysaccharide synthase activity or inducing a metabolic dormancy state (Ferreira et al., 2023; Bjorklund et al., 2020). In this study, the upregulation of oxidative stress response genes (such as *ahpC* and *sodA*) further supports this mechanism, while the compensatory antioxidant response of bacteria is clearly insufficient to counteract the metabolic disorder induced by SKN.

Moreover, amino acid metabolism, such as histidine and biotin, has been demonstrated to be closely associated with bacterial resistance and quorum sensing mechanisms (Tian et al., 2022; Miranda et al., 2019). SKN may disrupt the molecular basis of MRSA biofilm formation by inhibiting these metabolic pathways. Further research could explore the role of SKN in other critical metabolic pathways, such as carbon metabolism and energy metabolism, providing new perspectives for uncovering its multi-target regulatory mechanisms in MRSA biofilm formation.

Currently, MRSA infections primarily rely on vancomycin for treatment; however, the increasing prevalence of resistance poses significant challenges to therapeutic strategies (Orsi et al., 2011). SKN, as a natural product, has demonstrated promising antibiofilm activity, offering a novel direction for replacing or synergizing with antibiotics in treatment. Future research could further investigate its combined use with traditional antibiotics to achieve synergistic effects and delay the development of resistance. Existing studies have shown that plant-derived compounds, when used in combination with antibiotics, can significantly enhance antibacterial activity and reduce the rate of resistance development (Han et al., 2023; Sakarikou et al., 2020).

Despite this study revealing the anti-biofilm activity of SKN *in vitro* and in animal models, several limitations remain. Firstly, the omics analysis conducted in this research highlights the impact of SKN on specific metabolic pathways; however, these mechanisms require further validation through functional experiments. For instance, techniques such as metabolic flux analysis could be employed to track cysteine synthesis, consumption, and the rate of GSH conversion, or enzyme activity assays could be used to determine whether SKN directly inhibits the function of cysteine-metabolizing enzymes. Furthermore, the experimental results obtained from the rat model need to be substantiated through additional preclinical and clinical trials, particularly regarding the pharmacokinetics and safety profile of SKN in humans.

Future research could further investigate the impact of SKN on other metabolic pathways, such as carbon metabolism and energy metabolism, to elucidate its multifaceted mechanisms in regulating biofilm formation. Additionally, the combined application of SKN with existing antibiotics represents an important direction for future studies, particularly in clinical settings where combination therapy may enhance efficacy and delay the development of antibiotic resistance. Moreover, preclinical studies focusing on toxicity and pharmacokinetics will provide a solid foundation for the clinical application of SKN.

## Data Availability

The data presented in the study are deposited in the NCBI (SRA) repository, accession number PRJNA1423883.
